# Pharmaceutical 3D Printing Technology Integrating Nanomaterials and Nanodevices for Precision Neurological Therapies

**DOI:** 10.3390/pharmaceutics17030352

**Published:** 2025-03-09

**Authors:** Jurga Bernatoniene, Mindaugas Plieskis, Kestutis Petrikonis

**Affiliations:** 1 Department of Drug Technology and Social Pharmacy, Faculty of Pharmacy, Medical Academy, Lithuanian University of Health Sciences, Sukileliu pr. 13, LT-50161 Kaunas, Lithuania; 2 Institute of Pharmaceutical Technologies, Faculty of Pharmacy, Medical Academy, Lithuanian University of Health Sciences, Sukileliu pr. 13, LT-50161 Kaunas, Lithuania; 3 SwissHealix GmbH, Gutsch 21, Allenwinden, 6319 Zug, Switzerland; mindaugas.plieskis@swisshealix.ch; 4 Department of Neurology, Lithuanian University of Health Sciences, Eivenių str. 2, LT-50009 Kaunas, Lithuania; kestutis.petrikonis@lsmu.lt

**Keywords:** 3D pharmaceutical printing, nanomaterials, nanodevices, neurological diseases

## Abstract

Pharmaceutical 3D printing, combined with nanomaterials and nanodevices, presents a transformative approach to precision medicine for treating neurological diseases. This technology enables the creation of tailored dosage forms with controlled release profiles, enhancing drug delivery across the blood−brain barrier (BBB). The integration of nanoparticles, such as poly lactic-co-glycolic acid (PLGA), chitosan, and metallic nanomaterials, into 3D-printed scaffolds improves treatment efficacy by providing targeted and prolonged drug release. Recent advances have demonstrated the potential of these systems in treating conditions like Parkinson’s disease, epilepsy, and brain tumors. Moreover, 3D printing allows for multi-drug combinations and personalized formulations that adapt to individual patient needs. Novel drug delivery approaches, including stimuli-responsive systems, on-demand dosing, and theragnostics, provide new possibilities for the real-time monitoring and treatment of neurological disorders. Despite these innovations, challenges remain in terms of scalability, regulatory approval, and long-term safety. The future perspectives of this technology suggest its potential to revolutionize neurological treatments by offering patient-specific therapies, improved drug penetration, and enhanced treatment outcomes. This review discusses the current state, applications, and transformative potential of 3D printing and nanotechnology in neurological treatment, highlighting the need for further research to overcome the existing challenges.

## 1. Introduction

Recent advancements in three-dimensional (3D) printing technology have demonstrated its remarkable potential in pharmaceutical applications. The ability to produce pharmaceutical tablets that meet regulatory standards while matching the release profiles of commercially available products represents a significant breakthrough in 3D pharmaceutical printing, which enables the customized production of drug-loaded structures with precise control over the dose, shape, size, and drug release kinetics [1].

Various 3D printing techniques have been explored for creating tailored dosage forms for neurological conditions, each offering unique advantages in terms of drug delivery. Hot-melt extrusion (HME)-based fused deposition modeling (FDM) has gained significant attention for fabricating patient-specific medications, enabling continuous manufacturing processes for personalized therapies [2]. Inkjet printing and selective laser sintering (SLS) further expand the possibilities of customized drug formulations [2]. FDM prints drugs using hot-melt polymers for controlled release [3], while direct powder extrusion fuses drug powders with binders without solvents [4]. Semi-solid extrusion processes semi-solid materials, often incorporating hydrogels for sustained release [5]. SLS employs lasers to sinter powders, creating porous structures ideal for customized drug release [2], and binder jetting deposits liquid binders onto powder beds to form complex multi-drug systems with distinct release profiles [6,7]. With 3D printing, medications can be precisely formulated based on pharmacogenomics, age, weight, and disease progression, reducing adverse effects and improving therapeutic efficacy [8,9].

**Figure 1:**
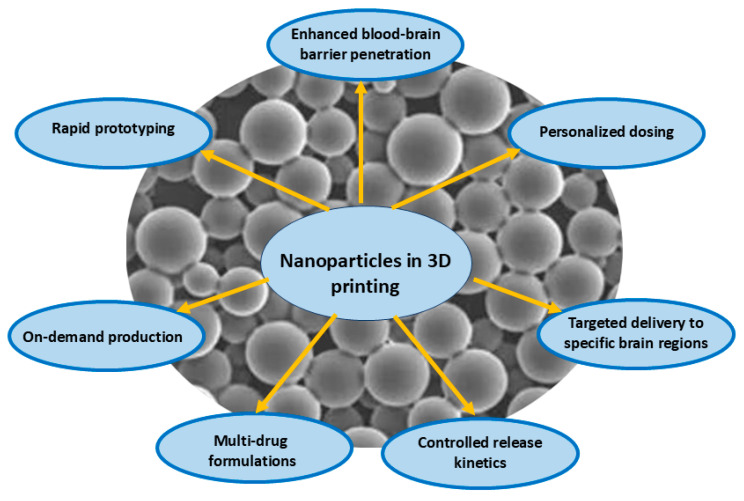
Advantages of the use of nanoparticles for 3D-printed pharmaceuticals [15,16,17,18,19].

The convergence of 3D printing technology with nanomaterials and nanodevices is transforming drug formulation and delivery, particularly for the treatment of neurological disorders [8,9]. This integration offers unprecedented control over dosage, release kinetics, and targeting specificity and promises to revolutionize precision medicine (Figure 1). However, the translation of these innovations from bench to bedside raises critical challenges, including scalability, safety, and regulatory issues [10,11,12].

Recent advances have shown that 3D-printed patient-specific dosage forms enable precise drug release tailored to individual needs [13,14]. The use of nanomaterials further enhances this by improving blood−brain barrier (BBB) penetration of drugs. For instance, nanoparticle-based systems increase brain drug uptake by 50% compared to conventional methods, addressing a significant limitation in neurological drug delivery [15].

A key question arises: how can pharmaceutical 3D printing combined with nanomaterials revolutionize neurological treatments, and what challenges need to be overcome to translate this from bench to bedside? Issues such as reproducibility in 3D printing [20,21,22] and regulatory gaps [11,12] are major obstacles. Long-term safety concerns regarding nanomaterial accumulation in the brain also require further investigation.

Moreover, the ability to fine-tune the release kinetics enables continuous or pulsatile drug release, which can be adapted to the unique needs of each neurological disorder. 3D printing allows for multi-drug delivery systems, combining therapies into a single dosage form, which is especially useful in managing complex conditions like epilepsy [17]. This precision also facilitates targeted delivery, improving therapeutic outcomes by concentrating treatment on specific brain regions affected by the disease [23]. Additionally, the role of 3D printing in neurosurgery is expanding, with patient-specific 3D-printed models improving surgical planning for intricate procedures [10]. As these technologies evolve, their integration into both therapeutic and diagnostic areas of neurology has the potential to redefine the management of these conditions.

This review explores the current state of 3D printing and nanotechnology in neurological treatments, addressing the challenges and opportunities of bringing these innovations to clinical practice.

## 2. Current State of 3D Printing Technology in Pharmaceuticals

Polymeric nanoparticles, such as those made from polylactic co-glycolic acid (PLGA) and chitosan, have gained significant attention for their role in controlled and targeted drug delivery systems [24,25,26]. These biodegradable polymers are ideal for creating sustained-release formulations that can improve drug efficacy and minimize side effects. Their integration into 3D-printed pharmaceutical forms has opened new pathways for personalized medicine, enabling precise spatial control of drug distribution [24,25,26]. This technology can produce complex structures tailored to specific anatomical needs, ensuring localized delivery and prolonged therapeutic effects. The process of embedding polymeric nanoparticles into 3D-printed scaffolds involves selecting materials compatible with the desired drug release profile. For instance, chitosan nanoparticles, with their excellent biocompatibility and mucoadhesive properties, can be incorporated into 3D-printed implants to enable localized and sustained drug release [27]. Moreover, polymeric nanoparticles have also been successfully integrated into 3D-printed drug delivery systems, demonstrating effective control over the release kinetics [2,28]. This integration also enables the simultaneous delivery of multiple drugs, which is a key advantage for complex treatments, such as cancer therapy [2,28].

In neurology, polymeric nanoparticles show significant potential in the treatment of neurological disorders like Parkinson’s disease [29,30,31,32,33], Alzheimer’s disease [34,35,36,37,38], and brain tumors [39,40,41,42,43,44,45]. The challenge of delivering drugs across the BBB has been a major limitation in neuropharmacology [42,43,44,45]. Researchers have demonstrated the ability of polymeric nanoparticles to cross the BBB when modified with targeting ligands or coated with surfactants [15,32]. When these nanoparticles are embedded into 3D-printed scaffolds, they can allow for localized and sustained drug delivery directly to the brain, improving drug efficacy and reducing systemic side effects [46,47]. Compared to traditional drug delivery systems, 3D-printed forms combined with polymeric nanoparticles offer enhanced precision [46]. Researchers have highlighted that, while challenges related to reproducibility and large-scale manufacturing remain, the integration of nanoparticles into 3D-printed scaffolds could revolutionize neurodegenerative disease treatment by providing more targeted and controlled drug release [48,49,50]. However, further research is needed to optimize these systems for clinical applications, especially in terms of patient safety and regulatory approval.

Metal-based nanomaterials, particularly gold, silver, and iron oxide nanoparticles, are emerging as key components in advanced drug delivery [34], especially when integrated with 3D printing for neurological applications [10,51]. Gold nanoparticles (AuNPs), known for their excellent biocompatibility and surface functionalization, are used in drug delivery and photothermal therapy applications [52,53,54,55]. AuNPs have been successfully integrated into 3D-printed scaffolds for bone regeneration [56] and myocardial tissue engineering [57], highlighting their potential for precision drug delivery and real-time monitoring [56]. Compared to polymeric nanoparticles, AuNPs provide superior diagnostic capabilities, which are crucial for neurological conditions that require constant observation [52,54].

Silver nanoparticles (AgNPs), which have strong antimicrobial properties, have shown promise in 3D-printed neurological implants, where infection control is critical [58]. Various studies have demonstrated their use in 3D-printed wound dressings, providing sustained antimicrobial activity and infection monitoring, an advantage over traditional polymeric systems, which lack theragnostic features [59,60,61,62]. In neurology, AgNPs can be applied to prevent infections in brain implants, enhancing recovery outcomes [63].

Iron oxide nanoparticles (IONPs) are particularly promising in neurology due to their magnetic properties, which facilitate targeted drug delivery across the BBB [64,65,66]. IONP-loaded 3D-printed scaffolds can be used for brain tumor therapy, offering both drug delivery and MRI imaging [66]. This is a major advancement over polymeric systems, as IONPs allow for precise targeting and real-time tracking of drug release, which is critical for treating neurological disorders [67,68,69,70].

**Figure 2:**
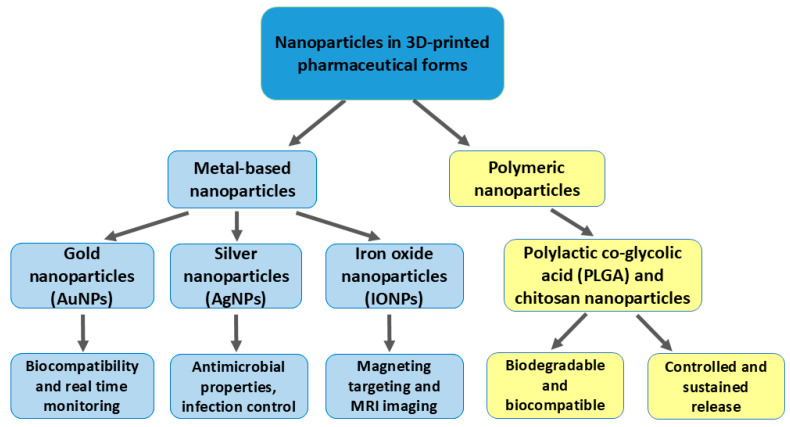
Integration of nanoparticles in 3D-printed pharmaceutical formulations: types and applications.

However, challenges remain in scaling up these technologies and achieving regulatory approval, especially for clinical applications in neurology [1]. The integration of both polymeric and metal-based nanomaterials into 3D-printed pharmaceutical forms presents significant advancements in drug delivery, particularly in neurological applications (Figure 2).

Polymeric nanoparticles, such as PLGA and chitosan, offer controlled, sustained release and the ability to cross the BBB, enhancing treatments for neurodegenerative diseases [24,25,26]. Metal-based nanoparticles, like gold, silver, and iron oxide, offer additional benefits through their diagnostic capabilities and targeting precision, as demonstrated in brain tumor therapies [10,51]. While gold nanoparticles enhance real-time monitoring, silver nanoparticles improve infection control in neurological implants [63]. Despite these advancements, challenges remain in large-scale production and regulatory approval, especially for complex neurological treatments.

Why is it beneficial to integrate nanoparticles into 3D pharmaceutical forms? It is important for targeted drug delivery and controlled release: nanoparticles allow drugs to be delivered precisely to specific body areas, reducing the risk of side effects [17]. It was demonstrated that integrating nanoparticles into 3D-printed pharmaceutical forms can help achieve precise control over drug release [26]. This is particularly advantageous in treatments such as cancer therapy, where localized and controlled release is crucial [39]. Furthermore, the integration of nanoparticles into 3D pharmaceutical forms is important for enhancing drug stability and efficacy. Nanoparticles can assist in crossing the BBB [15,32], which is a significant advantage in treating neurological diseases such as Parkinson’s and Alzheimer’s [30,31,34,38,47,54]. Nanoparticles improve the stability of drugs and allow active ingredients to remain in the body for longer durations, enhancing their therapeutic effect. Moreover, nanoparticles, when combined with 3D printing, enable the development of personalized drug forms tailored to individual patient needs [9,20]. These innovations allow for highly precise dosages and the creation of complex drug combinations, which are vital for treating challenging diseases [32]. By incorporating nanoparticles into 3D pharmaceutical forms, drug delivery can be made more efficient, safe, and better suited to individual patients, contributing to the advancement of personalized medicine and improving treatment outcomes across various medical fields.

## 3. Applications for Neurological Treatments

### 3.1. Nanodevices for Neurological Treatments: Comparative Insights

Nanotechnologies, particularly nanodevices integrated into 3D-printed pharmaceutical forms [71], are transforming neurological treatments by offering targeted drug delivery, real-time monitoring, and neurostimulation capabilities [51,63]. In the treatment of disorders such as Parkinson’s disease, Alzheimer’s disease, and brain tumors, traditional methods often face limitations, including difficulties in crossing the BBB, controlling drug release over time, and providing adequate monitoring [33]. Nanodevices address these challenges and open new horizons in personalized medicine [40].

One of the most promising applications of nanodevices in neurology is real-time monitoring and closed-loop drug delivery systems. In traditional treatments for Parkinson’s disease, for instance, patients often experience “on-off” effects, where drug efficacy fluctuates due to fixed dosing schedules that do not account for individual variations throughout the day [72]. These fluctuations can significantly impair the quality of life, as patients either do not receive enough medication or experience excessive dosing at different times. However, an approach with a closed-loop nanodevice system that continuously monitors dopamine levels and delivers dopamine-mimicking drugs only when necessary, thus reducing the “off” periods and extending the effective treatment duration from 40% to 75% of the day, highlights the potential of dynamic, patient-specific drug delivery systems [73]. Long-term epilepsy treatment faces challenges due to the BBB and inconsistent drug delivery [74]. A recent study evaluated a nanoengineered drug delivery system that enhances brain-targeted transport and enables the on-demand release of antiepileptic drugs through receptor-mediated transcytosis and photothermal BBB disruption [75]. It demonstrated efficient seizure inhibition with reduced drug dosage, rapid and sustained release, and favorable biosafety, offering a promising strategy for improving antiepileptic drug therapy [75].

The latest developments in 3D printing technology have further enhanced the functionality of nanodevices by enabling the fabrication of patient-specific drug delivery systems [76,77]. The ability to integrate nanomaterials into 3D-printed devices offers unprecedented precision in drug administration [78], particularly for the treatment of neurological disorders [9]. For instance, 3D-printed neural scaffolds loaded with nanodevices can be customized to fit a patient’s unique anatomical structure, providing highly localized and controlled drug release [58]. These developments underscore the transformative potential of combining 3D printing and nanotechnology to create personalized neurological therapies.

In comparison to polymeric nanoparticles, magnetic nanoparticles, particularly IONPs, offer additional functionalities, such as diagnostic imaging and drug delivery [64,65,66]. IONPs have been successfully integrated into 3D-printed scaffolds designed for brain tumor therapy [66]. These nanoparticles not only provided targeted drug delivery but also enabled real-time magnetic resonance imaging (MRI) to monitor tumor regression, a feature unavailable in traditional polymer-based systems. This dual functionality is essential for treating dynamic conditions like brain tumors, where ongoing monitoring is crucial. IONPs were shown to enhance drug concentration at the target site by up to 60%, compared to 30% when using polymeric nanoparticles like PLGA [67,68,69,70]. This level of precision and control underscores the superiority of magnetic nanoparticles in complex neurological treatments.

Additionally, infection control is a significant concern for neurological implants. Traditional polymeric nanoparticles do not possess intrinsic antimicrobial properties, making them less effective for long-term use in preventing infections [59,60,61,62]. AgNPs have demonstrated strong antimicrobial activity. AgNP-loaded 3D-printed wound dressings maintained antimicrobial properties for up to 30 days, in stark contrast to untreated dressings, which allowed bacterial growth within a week [79]. In the context of neurological implants, AgNPs can play a vital role in preventing post-surgical infections, enhancing recovery, and improving long-term outcomes.

The ability to control drug release over extended periods is another key advantage of the nanodevices. Polymeric nanoparticles, such as chitosan and PLGA, have been integrated into 3D-printed scaffolds to provide sustained drug release for up to 14 days [46]. This is particularly beneficial for the treatment of chronic neurological conditions, such as Parkinson’s disease, in which prolonged and controlled drug administration is critical. In contrast, metallic nanoparticles like AuNPs, although offering superior diagnostic capabilities, may not provide the same extended-release profiles but excel in areas such as photothermal therapy [55]. AuNPs, activated by infrared light, allow for the localized and precise treatment of cancerous cells, minimizing damage to surrounding healthy tissue—a feature lacking in polymeric systems [80,81,82].

The combination of nanoparticles and 3D-printed forms could revolutionize the treatment of neurodegenerative diseases by improving drug delivery across the BBB [83,84]. Nanodevices and nanomaterials integrated into 3D-printed pharmaceutical forms represent a major leap forward in the treatment of neurological diseases. By offering precision, controlled release, real-time monitoring, and infection control, these technologies promise to enhance patient outcomes and bring personalized medicine to the forefront of the field of neuropharmacology. However, continued research and development are needed to overcome challenges related to scalability, reproducibility, and regulatory approval. The future of neurological treatments lies in harnessing the full potential of nanotechnology to provide more effective and patient-tailored therapies.

Despite the many advantages of integrating nanomaterials into 3D-printed pharmaceutical forms, several challenges remain. Scaling these technologies for clinical applications is complex, as batch-to-batch variability in the manufacturing process can affect reproducibility, which is critical for regulatory approval [1,11,12,85]. Moreover, safety concerns regarding the long-term effects of nanomaterials, particularly metal-based nanomaterials, in sensitive areas like the brain need further investigation.

### 3.2. Personalized Dosage Forms for Neurological Disorders

**Figure 3:**
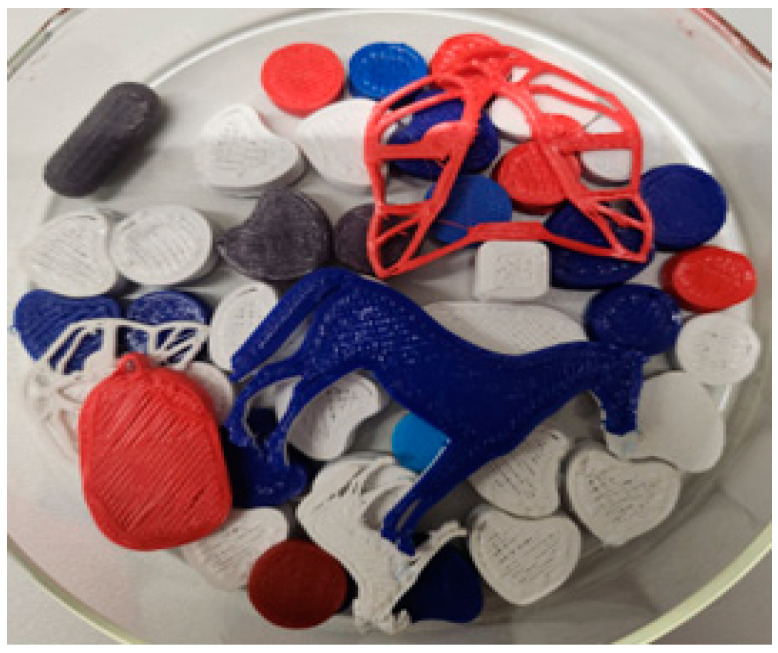
3D printed tablet forms.

The integration of 3D printing technology into pharmaceutical development presents a groundbreaking solution for personalized treatment, especially for neurological disorders, where precise dosing and complex drug combinations are often required [9]. Traditional pharmaceutical forms, such as tablets or capsules, typically offer standardized dosing that may not account for individual variations in patient metabolism, disease progression or drug interactions [1]. In contrast, 3D printing allows the customization of drug formulations and release profiles to suit specific patient needs, significantly enhancing treatment efficacy and patient compliance [86]. One of the primary advantages of 3D printing is its ability to combine multiple drugs into a single personalized dosage form, offering precise control over drug release [1,9,87]. Examples of 3D printed drug-loaded tablets are shown in Figure 3.

Neurological disorders, such as Parkinson’s disease, Alzheimer’s disease, epilepsy, and multiple sclerosis, require precise and personalized medication regimens due to the complexity of these conditions and the variability in patient responses [9,33]. The 3D printing of pharmaceuticals has revolutionized the production of personalized dosage forms, offering tailored drug release profiles, improved bioavailability, and enhanced patient adherence [22]. When combined with nanotechnology, these innovations open up possibilities for highly efficient and patient-centric treatments. In addition, 3D printing enables the co-delivery of multiple drugs, which is particularly beneficial for neurological disorders requiring polypharmacy, such as Parkinson’s disease and epilepsy [38,40]. Layer-by-layer production and the incorporation of polymers enable the development of sustained, delayed, or immediate-release formulations, optimizing drug bioavailability in the central nervous system [87].

Alternative solutions to 3D printing and nanotechnology for personalized neuropharmaceuticals that address key challenges, such as dose customization, drug stability, and controlled release, offer various innovative approaches to optimize drug delivery and patient adherence [88,89]. Microencapsulation involves enclosing drugs within protective polymeric shells, allowing controlled release, enhanced stability, and targeted delivery, which is particularly useful for neurological conditions requiring precise dosing [90,91]. Orodispersible films and tablets provide an effective solution for patients with swallowing difficulties, such as those with Parkinson’s disease, by allowing medications to dissolve quickly in the mouth and enhancing their absorption [92,93]. Additionally, liposome- and micelle-based drug carriers improve drug solubility, bioavailability, and distribution, facilitating more efficient delivery of neurological treatments [94,95]. Polymer-based hydrogels offer sustained and targeted drug release, which is particularly beneficial for transdermal patches or implantable systems that require prolonged therapeutic effects [96,97]. Bioadhesive drug delivery systems, which adhere to mucosal tissues such as the nasal lining, provide controlled drug release while improving patient compliance [98,99]. Lastly, electrospinning creates nanofiber-based drug carriers that allow precise control over drug loading and release kinetics, offering a scalable and adaptable alternative to additive manufacturing techniques [100,101,102].

The combination of 3D printing and nanotechnology holds immense promise for the future of personalized medicine in neurology. However, regulatory challenges, scalability issues, and cost concerns remain key barriers to their widespread clinical adoption. Further research is needed to optimize the materials, ensure safety, and develop standardized protocols for personalized neuropharmaceuticals [103].

### 3.3. Tailored Drug Release Profiles

One of the primary advantages of 3D-printed dosage forms is the ability to create complex drug release profiles tailored to individual patient needs [9,20]. This is particularly important in neurological disorders, where maintaining consistent drug levels is crucial for symptom control and preventing adverse effects of the drug. Traditional dosage forms often lack the flexibility needed to achieve this level of precision, leading to fluctuations in drug concentration that can worsen symptoms or cause side effects [22]. In contrast, 3D printing offers a way to customize both the timing and rate of drug release, providing more stable therapeutic levels [104].

**Figure 4:**
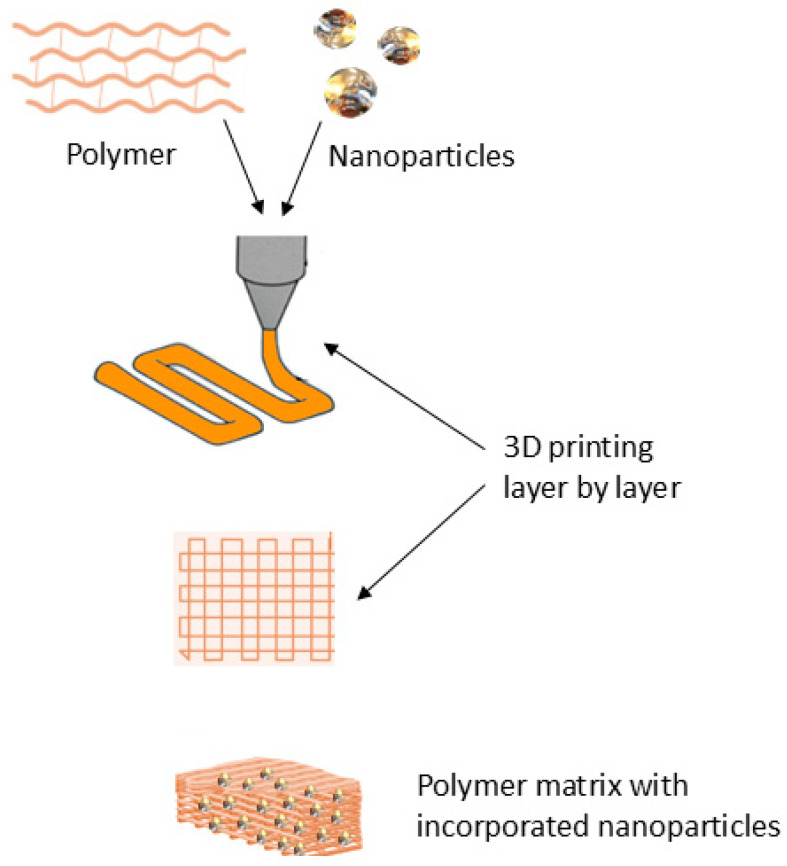
3D printing of polymer scaffolds containing nanoparticles.

The integration of nanotechnology into 3D-printed neurological drugs enhances drug solubility, stability, and targeted delivery, especially across the BBB [23]. Lipid-based nanoparticles (liposomes), polymeric nanoparticles, and nanocrystals can be incorporated into 3D-printed tablets or films to improve drug penetration into the CNS [15]. Nanoparticles responsive to pH, temperature, or enzymatic activity enable precision-controlled drug delivery, reducing systemic toxicity and enhancing therapeutic outcomes [105]. The use of biodegradable polymers, such as PLGA, in 3D-printed formulations ensures sustained drug release with minimal side effects [24,45,106]. An example of nanocomposite printing is shown in Figure 4.

For example, in the treatment of epilepsy, researchers have demonstrated the effectiveness of 3D-printed levetiracetam tablets in improving pharmacokinetic profiles compared with conventional formulations. By utilizing semi-solid extrusion (SSE) printing, the tablets were designed with internal structures that modulated drug release by adjusting the infill percentage and pattern [107]. This allowed for a more stable release of the drug throughout the day, reducing seizure frequency and improving patient outcomes (Table 1). Such fine control over drug release profiles is critical in epilepsy, where maintaining steady drug levels can significantly impact the management of seizures.

Furthermore, another study utilized FDM 3D printing to develop a floating sustained-release system containing pregabalin, using hypromellose acetate succinate and polyethylene glycol as excipients [110]. The optimized formulation demonstrated zero-order drug release, with the stability and crystallinity of the drug maintained after printing, highlighting the potential of FDM for creating personalized floating dosage forms with controlled release profiles. Hydroxypropyl-β-cyclodextrin was also used to 3D print orodispersible and immediate-release printlets of poorly soluble carbamazepine via semi-solid extrusion, with adjustments in cellulose ether composition to regulate drug release. The results demonstrated the feasibility of forming carbamazepine-HPβCD complexes in situ, producing printlets with desirable physical properties and tunable drug release profiles for oral delivery. Similarly, for Parkinson’s disease, precise control of dopamine levels is essential for managing motor symptoms effectively. Extended-release 3D-printed pramipexole tablets were prepared using fused deposition modeling (FDM) to enable personalized dosing for Parkinson’s disease, including commercially available and intermediate doses not found in the market [112]. The optimized 3D tablet formulation demonstrated stability, similar pharmacokinetic profiles, and a relative bioavailability of 107.6% compared to that of the marketed tablet, highlighting FDM-3D printing’s potential for creating personalized, extended-release dosage forms [112]. The ability to customize drug release timing is further enhanced by the integration of polymeric nanoparticles, such as PLGA and chitosan, into 3D-printed forms. Incorporating these nanoparticles into 3D-printed dosage forms extended drug release up to 14 days, a notable improvement over traditional forms, which generally provide sustained release for only 3–5 days [46]. This is particularly beneficial in chronic conditions like Parkinson’s disease, where consistent drug levels are critical for managing symptoms and reducing complications from pulsative dopamine receptor activation and fluctuating doses.

The management of Alzheimer’s disease focuses on alleviating symptoms and slowing disease progression through pharmacological interventions with cholinesterase inhibitors, NMDA receptor antagonists, or therapies targeting amyloid-beta [29]. Digital light processing (DLP) and semi-solid extrusion (SSE) 3D printing techniques were used to produce donepezil-coated microneedle patches for transdermal delivery in Alzheimer’s disease treatment. The microneedles demonstrated successful drug incorporation, mechanical robustness, enhanced skin permeation, sustained drug release, and biocompatibility, highlighting their potential for clinical transdermal applications [113]. In another study, curcumin-loaded PLGA nanoparticles were embedded in 3D-printed sodium alginate/gelatin scaffolds for sublingual delivery to treat Alzheimer’s disease, achieving controlled drug release for 18 days [114].

Nanoparticle-loaded 3D-printed forms can improve drug penetration into brain tissues [115]. The ability to deliver drugs directly to the brain while controlling the release rate could reduce systemic side effects and enhance treatment efficacy [23,32,38]. With continued advancements, 3D-printed nanomedicine could revolutionize the management of neurological disorders, offering patients safer, more effective, and precisely tailored treatment options.

### 3.4. Multi-Drug Combinations

Neurological conditions like Alzheimer’s disease, Parkinson’s disease, and multiple sclerosis often require combination therapies involving multiple medications [9,10]. Traditional manufacturing methods face significant challenges in combining drugs with different properties and release requirements into a single dosage form, leading to reduced patient adherence [9]. 3D printing overcomes these limitations by enabling the precise incorporation of multiple drugs into a single form, allowing tailored doses and release profiles [9].

For epilepsy treatment, binder jet 3D printing (BJ-3DP) was used to develop high-precision, multicompartmental, and dispersible tablets containing levetiracetam and pyridoxine hydrochloride, achieving accurate dose control through layered printing and overcoming drug migration issues with modified drying methods. The resulting tablets demonstrated strong mechanical properties, rapid disintegration, and efficient drug release [116] (Table 2).

FDM 3D-printing has been applied to develop personalized therapy for Parkinson’s disease, creating floating mini-polypills with variable dosages of levodopa, benserazide, and pramipexole for both rapid and prolonged drug release to enhance levodopa absorption in the upper gastrointestinal tract, improve patient compliance with swallowing difficulties, and allow for individualized, sustained drug delivery [117]. This flexibility allows for more tailored treatment regimens, potentially improving symptom control throughout the day and reducing the need for multiple doses (Table 2).

For Parkinson’s disease, a 3D-printed tablet formulation was developed for levodopa/carbidopa using direct powder extrusion, aiming for rapid drug release and integration into closed-loop medication management systems in hospital pharmacies. The tablets demonstrated high dose accuracy, immediate levodopa release, strong physical stability, and suitability for hospital settings [118,119], although carbidopa is unstable during the printing process [118].

3D printing enables the precise combination of multiple drugs into a single dosage form, optimizing pharmacokinetics and improving patient adherence. Compared to traditional multi-pill regimens, 3D-printed forms show superior results in maintaining drug concentrations, reducing side effects, and increasing treatment adherence by 20–30%, particularly for complex neurological conditions [22].

### 3.5. Dose Adjustment Based on the Response of Patients

The flexibility offered by 3D printing in drug manufacturing allows for rapid and precise dose adjustments based on patient responses, which is particularly valuable in managing neurological disorders, where titration is often essential [1,9,22]. Traditional drug manufacturing processes can be slow to adapt to dose modifications, leading to treatment gaps or imprecise methods, such as tablet splitting. In contrast, 3D printing allows for individualized dose adjustments without altering the size or appearance of a tablet, offering a more seamless and patient-friendly solution [78].

3D-printed tablets have been produced with varying doses of pregabalin, a common drug used for neuropathic pain [120]. They can be made with consistent physical characteristics while allowing for different drug loadings, ensuring more precise dose titration without the need for new prescriptions or multiple forms. This stands in contrast to traditional methods, where dose flexibility is often limited to fixed strengths, requiring patients to split tablets or take multiple pills, which can lead to dosage errors and decreased adherence [120].

In comparison, conventional approaches often rely on standardized dosages that may not meet the personalized needs of patients [17]. 3D printing technology can easily modify drug dosages to suit specific therapeutic requirements [17], which is particularly important in conditions like epilepsy or Parkinson’s disease, where even small variations in dosage can dramatically affect symptom control. The ability to adjust doses without changing the overall tablet shape or size ensures that patients are not confused by frequent changes in the medication appearance, which is a common issue in traditional dose adjustment strategies [17].

Furthermore, the advantage of 3D printing for dose adjustment is particularly evident in pediatric neurology. Traditional methods for adjusting dosages in children often involve splitting tablets or using liquid formulations, which can be inaccurate and lead to inconsistent dosing [121]. In contrast, 3D-printed dosage forms allow precise dose customization based on individual factors, such as weight and response. This level of precision has been shown to improve treatment outcomes by reducing side effects and ensuring optimal drug exposure [122]. Children with epilepsy have benefited from this tailored approach, achieving better symptom control with fewer side effects compared to conventional therapies [123].

In addition to rapid dose adjustments, 3D printing allows the incorporation of multiple release profiles within a single tablet, further improving treatment flexibility. Multi-layered 3D-printed tablets can deliver both immediate and sustained drug release [124]. This allows for on-demand dose adjustments while maintaining long-term therapeutic effects, providing a more holistic and personalized treatment plan. In comparison, traditional manufacturing requires multiple pills or combination therapies, which can reduce patient compliance and increase the complexity of treatment regimens [124].

Overall, the comparison between traditional and 3D-printed dose adjustment methods highlights the superiority of 3D printing in achieving precise and personalized treatment. The flexibility it offers in dose titration, especially for sensitive neurological conditions, ensures better patient outcomes, increased adherence, and reduced medication errors [22]. As research continues to validate these approaches, 3D printing may become a standard practice in personalized neurology treatment.

## 4. Innovative Drug Delivery Systems

**Figure 5:**
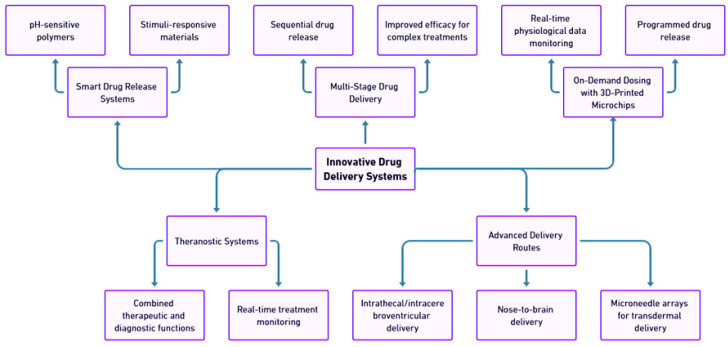
Innovative drug delivery systems.

In recent years, the focus of drug delivery systems has shifted toward more sophisticated and adaptable technologies (Figure 5). The intersection of 3D printing, microfabrication, and smart materials has led to the development of highly innovative delivery platforms, particularly for neurological treatments [10,47,85,115].

Unlike traditional methods, these new approaches focus not only on sustained drug release but also on the triggered release, multi-stage drug delivery, and on-demand dosing, adapting to real-time patient needs and specific neurological challenges [10,47,85,115].

### 4.1. Smart Drug Release Systems

Smart materials incorporated into 3D-printed devices enable the creation of *stimuli-responsive* drug delivery systems that can release their payloads in response to specific triggers, such as pH and temperature, and external stimuli like magnetic fields or light [125,126]. For instance, pH-sensitive polymers used in 3D-printed scaffolds can ensure that drugs are released only when a target area, such as inflamed neural tissue, reaches a certain acidity level [126]. Recently, an injectable pH thermo-responsive nanocellulose/chitosan-based hydrogel scaffold was created for neural stem cell encapsulation and delivery in glioblastoma multiforme treatment, demonstrating that cell incorporation did not significantly alter scaffold properties, while cellulose nanocrystals prolonged degradation at higher cell densities [127]. The hydrogels supported high stem cell viability, sustained therapeutic tumor necrosis factor-related apoptosis-inducing ligand release, and effective tumor cell killing, highlighting their potential as a promising local cell delivery system for post-surgical glioblastoma treatment [127].

### 4.2. Multi-Stage Drug Delivery

Another emerging concept is multi-stage delivery, in which a single 3D-printed device is designed to release different drugs or therapeutic agents at distinct stages [128]. This approach is particularly beneficial for diseases that require multi-step treatment, such as brain tumors, where both chemotherapy and radioprotective agents need to be delivered sequentially [129]. Studies have shown that multi-stage systems can improve treatment efficacy by up to 40% compared to simultaneous delivery, as sequential dosing allows for optimized drug bioavailability and timing [78]. These systems made possible through 3D printing, can also address drug resistance, a common issue in the long-term treatment of neurological diseases [9].

### 4.3. On-Demand Dosing with 3D-Printed Microchips

In addition to environmental stimuli, *on-demand dosing* via 3D-printed microchips is an innovative leap in neurological drug delivery [130]. These microchips, often embedded with nanodevices, can be programmed to release drugs based on real-time physiological data from patients [72,73]. For example, nanosensors in the brain can monitor dopamine levels in patients with Parkinson’s disease and activate drug release when dopamine levels drop below a critical threshold [73], allowing for real-time, precise management of symptoms without the need for patient intervention. Such systems could lead to a 50% improvement in symptom management for patients with fluctuating dopamine levels compared to fixed dosing regimens [73].

### 4.4. Theragnostic Systems for Real-Time Monitoring

Beyond drug delivery, the combination of 3D printing and nanotechnology has enabled the creation of *theragnostic* platforms, which are devices that combine therapeutic and diagnostic functions [70]. These systems allow real-time monitoring of the disease state while simultaneously delivering treatment. For instance, iron oxide nanoparticles integrated into 3D-printed devices can provide MRI imaging capabilities while delivering chemotherapy drugs directly to brain tumors [67,68,69,70]. This dual-function approach led to a 35% improvement in tumor reduction compared to traditional chemotherapy while also allowing to monitor the treatment in real-time [69].

### 4.5. Intrathecal and Intracerebroventricular Delivery

One of the most significant challenges in treating central nervous system disorders is the delivery of drugs across the BBB [23]. 3D printing can be used to create customized implants for intrathecal or intracerebroventricular drug delivery, providing a direct route to the central nervous system [47].

Researchers have developed 3D-printed polymeric implants loaded with chemotherapeutic agents for the treatment of glioblastoma [66,68,69]. These implants allow sustained local drug delivery while minimizing systemic side effects. The 3D printing process enables precise control over the implant geometry and internal structure, which in turn influences the drug release kinetics. By optimizing these parameters, researchers have achieved sustained drug release over several weeks, potentially improving treatment efficacy and reducing the need for repeated, invasive procedures [66,68,69].

Similar approaches are being explored for other neurological conditions. For instance, 3D-printed intrathecal delivery systems are being investigated for the treatment of chronic pain and spasticity associated with conditions like multiple sclerosis [131]. These systems could provide more consistent drug levels in the cerebrospinal fluid, potentially improving symptom control and reducing side effects compared to oral medications.

### 4.6. Nose-to-Brain Delivery

The nose-to-brain pathway presents a non-invasive and efficient route for delivering drugs directly to the central nervous system (CNS), bypassing the BBB [132]. This pathway, which leverages the olfactory and trigeminal nerves, offers a promising alternative to systemic drug delivery, which is often limited by poor BBB penetration [132,133]. The application of 3D printing in developing intranasal delivery devices has opened new possibilities for improving drug deposition, retention, and controlled release in the nasal cavity, leading to more effective CNS drug delivery [134].

A cell-based nasal model using a 3D-printed human nasal cavity replica was developed, where PLGA nanoparticles were aerosolized into the 3D-printed nasal cavity, which included an insert of air–liquid interface RPMI 2650 cells positioned in the olfactory region, to assess the deposition and potential for nose-to-brain drug delivery [135]. Additionally, the use of 3D printing for fabricating nasal stents has been explored, where the incorporation of nanoparticles, such as halloysite clay, into the polymer material can enhance properties like adhesion, tensile strength, and thermal stability [136]. This approach suggests the potential for creating customized nasal devices with improved mechanical and bioactive properties [136].

A 40% increase in the brain uptake of rivastigmine for Alzheimer’s disease was reported when using 3D-printed nasal inserts loaded with nanoparticles compared to traditional nasal spray formulations [137]. These 3D-printed inserts were specifically designed to maximize surface contact with the nasal mucosa, optimizing drug absorption. Similarly, it was reported that conventional nasal sprays often suffer from rapid drug clearance and suboptimal deposition, limiting their effectiveness in delivering drugs to the brain [138]. The precise control over geometry and surface properties provided by 3D printing addresses this limitation by improving the drug residence time in the nasal cavity, thereby enhancing drug bioavailability [133].

Further supporting the potential of nose-to-brain delivery, the challenges of traditional methods in overcoming the BBB were reviewed, noting that less than 2% of systemically administered drugs reach the brain [139]. In contrast, studies using 3D-printed nasal devices have shown significant improvements in CNS drug concentrations. A 50% increase in rivastigmine bioavailability in the brain compared to oral administration has been reported, reducing the need for high systemic doses that often lead to adverse side effects [137].

The delivery of dopamine directly to the brain resulted in an improvement in symptom management compared to oral levodopa in Parkinson’s disease [140]. This improvement is particularly relevant given that oral formulations are often subject to first-pass metabolism, leading to fluctuating drug levels and inconsistent therapeutic effects. The use of 3D-printed devices ensures more stable and consistent drug delivery directly to the CNS, providing a smoother symptom management profile [141].

These studies highlight the innovative application of 3D printing technology combined with nanoparticle integration to develop advanced nasal delivery systems aiming to improve therapeutic outcomes for various medical conditions.

### 4.7. Microneedle Arrays for Transdermal Delivery

**Figure 6:**
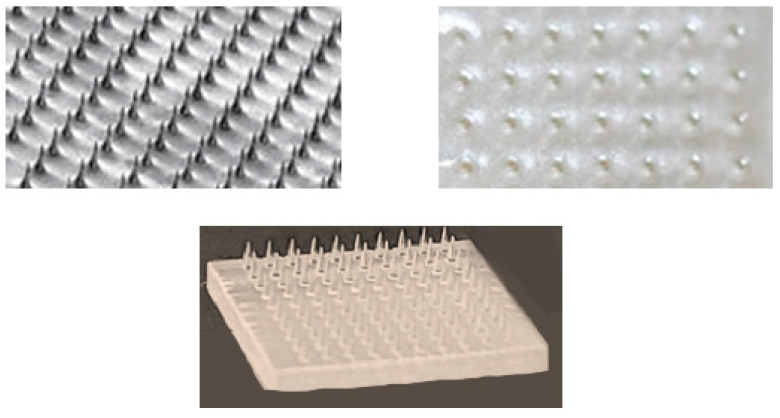
Examples of microneedle patches.

3D-printed microneedle arrays offer a groundbreaking approach to transdermal drug delivery, particularly for neurological conditions (Figure 6).

This technology enables the precise, minimally invasive delivery of drugs across the skin barrier, offering improved bioavailability and consistency compared to oral or injectable routes [142,143,144,145]. Unlike traditional methods, microneedle arrays can be engineered to accommodate drugs that are otherwise difficult to deliver due to poor skin permeability, enhancing therapeutic efficacy [142,145,146].

One key advantage of microneedles is their ability to customize drug penetration and release profiles [147,148]. An analgesic microneedle patch has been developed using dissolvable microneedles to transdermally deliver selective calcitonin gene-related peptide, which is a neuropeptide released from sensory nerve endings [149]. The analgesic microneedle patches provided effective, targeted analgesia in rat models without affecting normal sensory or motor functions, showing advantages over conventional therapies with no skin irritation or systemic side effects [149]. A study formulated lacosamide-loaded polymeric microneedles using carboxymethyl cellulose and Eudragit S 100, aiming to enhance brain delivery via the nasal route and overcome the BBB. The results indicated that Eudragit S 100-based microneedles extended drug release up to 96 h, suggesting their potential for improved epilepsy treatment [150]. Donepezil-coated microneedle patches for transdermal delivery in Alzheimer’s disease treatment were fabricated using digital light processing (DLP) and semi-solid extrusion (SSE) 3D printing techniques. These microneedles exhibited effective drug incorporation, strong mechanical properties, improved skin permeation, sustained drug release, and biocompatibility, emphasizing their potential for clinical transdermal applications [113].

Multi-drug delivery is another potential advantage of 3D-printed microneedles. Unlike conventional transdermal patches, which often deliver a single active ingredient, microneedles can be designed to release multiple drugs at varied release rates [147,148]. One study introduced an integrated theranostic microneedle array system employing an array of colorimetric sensors to quantitatively measure pH, glucose, and lactate levels. This system also featured a remotely triggered mechanism enabling on-demand drug delivery, showcasing the capability of 3D-printed microneedles in combining diagnostics with multi-drug therapeutic applications [151].

Another key benefit is personalization. Patients with conditions affecting skin permeability, such as diabetes, dermatitis, or psoriasis, often face challenges in drug absorption through the skin [152]. One study demonstrated the development of 3D-printed microneedles with varied needle lengths that can be tailored to penetrate specific skin depths [153]. This approach could improve transdermal drug delivery in individuals with skin conditions that affect the permeability.

In addition to tailoring the microneedle design, integration with nanoparticle technology offers an additional layer of innovation [146]. For instance, a study introduced all-inkjet-printed conductive microneedles based on silver nanoparticles and demonstrated a scalable fabrication method for in vivo biosensing applications [154]. Many neurological drugs, such as large peptides and proteins, face significant barriers to effective transdermal delivery [155]. Additionally, even if a drug permeates the skin layers, the BBB poses another significant obstacle for neurological therapeutics, restricting the entry of large-molecule neurotherapeutics and over 98% of small-molecule drugs [84]. This dual-layered defense system complicates the delivery of neurological drugs via transdermal methods. Nanoparticles can be incorporated into microneedles to encapsulate drugs, improving skin permeability and drug bioavailability [156].

These innovations hold promise for conditions where personalized and minimally invasive drug delivery is critical. Future research is likely to expand the applications of this technology, leading to more effective and patient-friendly treatment options for complex neurological disorders.

## 5. Future Perspectives and Transformative Potential

The convergence of pharmaceutical 3D printing technology with nanomaterials and nanodevices holds significant promise for revolutionizing neurological treatment [9,58]. This intersection is poised to advance the field of precision medicine for neurological disorders, enabling the development of more adaptive and patient-specific therapies [157]. In this section, we explore several transformative developments on the horizon and discuss their potential impact.

### 5.1. Adaptive Neural Interfaces

The integration of 3D-printed scaffolds with nanoelectronic components may pave the way for adaptive neural interfaces capable of dynamic responses based on real-time neural activity [158]. A promising application lies in creating 3D-printed neural meshes embedded with nanoscale sensors and actuators that can monitor local neurotransmitter levels [159] and adjust drug release accordingly. Such interfaces could provide highly personalized treatments for conditions like Parkinson’s disease and epilepsy. However, it remains to be seen how these interfaces will handle the complexities of neural feedback in real-time and whether they can maintain long-term functionality without causing adverse effects.

### 5.2. Biomimetic Brain-on-a-Chip Models

Advances in 3D bioprinting and nanoengineering are driving the development of sophisticated brain-on-a-chip models [160]. These in vitro systems can replicate complex neural networks with remarkable accuracy by incorporating various cell types, extracellular matrices, and functional vasculature [161]. While these models hold tremendous potential for drug screening, personalized medicine, and neurodegenerative disease research, the challenge lies in achieving a level of biological mimicry that fully captures the in vivo environment [162,163]. Further research is needed to validate these models’ predictive power and explore their role in understanding the intricate mechanisms of neurological diseases.

### 5.3. Nanoscale Neuroregeneration Platforms

The integration of 3D-printed scaffolds and nanomaterials could lead to breakthroughs in neural regeneration therapies [51,58]. For instance, the development of 3D-printed hydrogels with embedded nanofibers and growth factor-releasing nanoparticles can create optimal conditions for nerve regeneration post-injury. These scaffolds can be tailored to a patient’s anatomy and are designed to biodegrade as new neural tissues are formed [51,58]. The success of these platforms, however, depends on how well they can be personalized to diverse patient anatomies and whether they can sustain the regeneration process over the long term without inducing immune responses.

Hydrogels are promising systems for the 3D printing of nanoscale pharmaceutics, offering sustained and targeted delivery of nanoparticles and extracellular vesicles for various clinical applications [164]. Their biocompatible structure ensures prolonged retention at the target site, controlled therapeutic release, and potential for on-demand drug delivery through stimuli-responsive components [77,164]. This approach enhances therapeutic efficacy, minimizes toxicity, and holds significant potential for application in tissue regeneration and cancer treatment [77].

In neurological disorders, hydrogels are used for nerve regeneration [164]. A novel strategy was developed for repairing peripheral nerve injury using a nerve guidance conduit (NGC) composed of AuNPs and brain-derived neurotrophic factor-encapsulated chitosan within a laminin-coated nanofiber of PLGA conduit, combined with rat adipose-derived stem cells suspended in alginate [165].

In vivo experiments using a rat sciatic nerve transection model demonstrated that this combination synergistically enhanced nerve regeneration. Histological, immunohistochemical, and behavioral analyses confirmed that AuNPs and brain-derived neurotrophic factor-encapsulated chitosan in NGC significantly improved the regenerative performance of rat adipose-derived stem cells, suggesting a promising new approach for peripheral nerve repair [165]. Another study investigated a chitin-based NGC incorporating conductive poly(3,4-ethylenedioxythiophene) nanoparticles (PEDOT NPs) and a cell-adhesive tetrapeptide to promote sciatic nerve regeneration [166]. Partial deacetylation of chitin enhanced electrostatic interactions with PEDOT strengthened the hydrogel and provided active sites for peptide modification, leading to improved Schwann cell adhesion, proliferation, and expression of nerve regeneration-related genes. In vivo experiments in a rat sciatic nerve defect model demonstrated that the chitin/PEDOT composite facilitated nerve regeneration comparable to autografts, highlighting its potential as an effective material for peripheral nerve repair [166].

Furthermore, a study investigated the potential of berberine-loaded chitosan nanoparticles within an alginate-chitosan hydrogel scaffold for spinal cord injury repair [167]. Physicochemical analyses and in vivo experiments using hemisected spinal cord injury rats demonstrated that the scaffold, especially when seeded with endometrial stem cells, promoted cell adhesion, survival, and enhanced neuronal growth marker (neurofilament) expression. Behavioral assessments using the BBB test confirmed improved sensory and motor function recovery, suggesting that this combination therapy holds promise for spinal cord regeneration and reducing secondary damage [167].

### 5.4. Quantum Dot-Enhanced Theragnostics

Integrating quantum dots into 3D-printed drug delivery systems could revolutionize theragnostics for neurological disorders [71]. These nanostructures, acting both as imaging agents and drug carriers, allow for real-time monitoring of drug distribution and efficacy in the brain [168,169]. This approach could significantly improve treatments for brain tumors, enabling precise visualization and targeted therapy [170].

Recently, carbon quantum dots have emerged as promising candidates for targeted drug delivery, owing to their ultrafine size, versatile surface functional groups, and exceptional sensitivity to pH, temperature, and light—enabling them to effectively penetrate challenging barriers like the blood–tumor and blood–brain barriers [171]. However, questions regarding the safety and long-term biocompatibility of quantum dots in the neural environment need thorough investigation before they can be clinically implemented.

### 5.5. AI-Driven Personalized Formulations

In the future, artificial intelligence (AI) algorithms may be used to optimize 3D-printed formulations based on individual patient data, including genetic profiles, neuroimaging results, and biosensor feedback [172,173]. Such technology could lead to evolving treatment regimens that adapt to the patient’s changing neurological status, maximizing efficacy while minimizing side effects. The practical application of AI-driven formulations, however, requires a comprehensive understanding of the regulatory challenges and ethical considerations surrounding the use of AI in patient care [172,173].

### 5.6. Nanorobot-Assisted Neurosurgery

3D printing has the potential to facilitate the production of customized nanorobots for minimally invasive neurosurgical procedures. These nanodevices, directed by external magnetic fields or autonomous navigation systems, can enable targeted drug delivery, plaque removal in Alzheimer’s disease, or precise tumor ablation while sparing healthy tissue [174,175]. A critical discussion point among researchers is the safety of deploying these nanorobots in the brain’s complex environment and the need for real-time monitoring systems to ensure accuracy and avoid unintended tissue damage [174,175].

### 5.7. Neurovascular Remodeling Therapies

The development of advanced 3D-printed constructs incorporating angiogenic nanoparticles and neural stem cells presents new opportunities for stroke recovery [51,176,177]. These constructs, designed to match a patient’s lesion geometry, can promote vascular and neural regeneration, potentially restoring lost function. However, researchers face the challenge of fine-tuning the interaction between these materials and host tissue to optimize repair while minimizing inflammation or rejection [51,176,177].

### 5.8. Epigenetic Modulation Platforms

Emerging 3D-printed nanodevices capable of targeted epigenetic modulation could offer novel treatment avenues for neurodevelopmental [178] and psychiatric disorders [179]. By delivering epigenetic modifiers to specific brain regions, these devices could potentially reverse the abnormal gene expression patterns associated with conditions such as autism and schizophrenia [178,179]. While promising, this approach raises concerns regarding the specificity and permanence of epigenetic modifications, necessitating extensive investigations to determine its feasibility and safety.

### 5.9. Neuroplasticity-Enhancing Hybrid Systems

The combination of 3D-printed hybrid systems with electrical stimulation and controlled release of neuroplasticity-enhancing drugs could significantly improve rehabilitation outcomes in patients with traumatic brain injuries or stroke [51,58]. Tailored to target specific neural circuits, these systems may optimize the brain’s natural repair mechanisms [51]. However, questions remain regarding the optimal design and stimulation parameters for achieving desired therapeutic outcomes across different patient populations [51,58].

Utilizing the precision of 3D printing and the responsiveness of nanomaterials, future drug delivery systems can be synchronized with the body’s circadian rhythms. This chronotherapeutic approach could enhance the treatment efficacy for conditions influenced by circadian cycles, such as sleep and mood disorders [180,181]. The success of these systems hinges on the accuracy of circadian rhythm monitoring and their ability to adapt to individual variability in circadian patterns.

### 5.10. Challenges of 3D Pharmaceutical Printing Using Nanomaterials

3D pharmaceutical printing with nanomaterials presents several challenges that must be addressed to ensure safe and effective application [104,182,183,184]. One primary concern is the potential toxicity of certain nanomaterials, which may render them unsuitable for pharmaceutical and medical use [104,184]. Additionally, achieving uniform dispersion of nanoparticles within the printing medium is difficult; agglomeration can lead to defects and inconsistencies in the final product [104,184]. The high surface area of nanomaterials often results in increased reactivity, posing safety hazards such as health risks upon inhalation [184]. Scaling up nanomaterial production while maintaining consistent quality remains problematic, as material properties can change when transitioning from the nanoscale to bulk quantities [104]. Moreover, precise control of the printing parameters is crucial for preventing residual stresses and microstructural defects, which can compromise the integrity of the printed objects. Addressing these issues requires advancements in materials science, improved safety protocols, and the development of standardized manufacturing processes [104,184].

### 5.11. Regulatory Framework for Nanomaterials and 3D Printing in Pharmaceutical Manufacturing

The U.S. Food and Drug Administration (FDA) has issued a guidance document, “Drug Products, Including Biological Products, that Contain Nanomaterials”, providing recommendations for the development of human drugs and biological products containing nanomaterials [185]. This guidance addresses the considerations related to the characterization, manufacturing, and evaluation of such products to ensure their quality, safety, and efficacy. It emphasizes a risk-based approach and highlights the importance of understanding the unique properties of nanomaterials for drug development [185].

In January 2025, the European Medicines Agency (EMA) issued a report titled “Nanotechnology-based medicinal products for human use”, presenting a comprehensive overview of the current landscape and future prospects of nanotechnology in medicine [186]. This document is part of the EU Innovation Network (EU-IN) Horizon Scanning Reports, which aim to identify and analyze emerging technologies with a significant potential impact on public health [186].

In 2017, the FDA released the Technical Considerations for Additive Manufactured Medical Devices guidance, outlining recommendations for device development, validation, manufacturing, testing, and labeling. This guidance also extends to the use of 3D printing in hospitals, medical centers, and pharmacies [187]. In December 2021, the FDA issued a discussion paper proposing future draft and final guidelines based on stakeholder feedback [187]. While there have been suggestions for the FDA to establish formal standards for assessing device risks, it has not yet issued specific regulations for 3D-printed pharmaceuticals [187,188].

The EMA also has not yet introduced dedicated guidelines for 3D-printed drug formulations [11]. Manufacturers utilizing 3D printing for medical devices and pharmaceuticals must conduct risk assessments to comply with the EU Machinery Directive 2006/42/EC [189]. However, the EU regulatory framework remains technologically neutral, allowing flexibility in technical approaches while ensuring compliance with health and safety standards [188].

Thus, both the FDA and EMA have taken significant steps to address the regulatory landscape of nanotechnology-based and 3D-printed medicinal products. However, despite these advancements, coordinated efforts between regulatory agencies are essential to establish clear and standardized guidelines to ensure the safe and effective development of emerging pharmaceutical technologies.

## 6. Conclusions

Pharmaceutical 3D printing technology, when combined with nanomaterials and nanodevices, shows great promise for advancing precision medicine for neurological diseases. This integration enables tailored dosage forms, improved drug delivery across the BBB, and multi-drug combinations, offering personalized treatment options for complex conditions like Parkinson’s disease, epilepsy, and brain tumors. While advances in nanoparticle-enhanced 3D-printed systems have demonstrated improved therapeutic outcomes, challenges related to scalability, regulatory approval, and long-term safety need to be addressed. Additionally, innovations such as stimuli-responsive systems and AI-driven formulations highlight the potential for adaptive and real-time patient-specific treatments. Despite these obstacles, continued research and technological development in this field can revolutionize neuropharmacology, offering more effective and individualized therapies. Ultimately, the future of neurological treatment lies in optimizing these 3D-printed nanoparticle-integrated systems to enhance patient care and quality of life.

## Figures and Tables

**Table 1:** Examples of 3D-printed tailored mono-drug release profiles for neurological disorders.

Neurological Condition	Drug	3D Printing Technique	Key Benefits
**Epilepsy**	Levetiracetam	Semi-solid extrusion (SSE)	Improved pharmacokinetic profiles; reduced seizure frequency [107].
		SSE	Immediate release, on- demand printing [108].
		Modified binder jetting	Spritam, first FDA approved 3D-printed drug, flash-dispersing [109].
	Pregabalin	Fused deposition modeling (FDM)	Floating sustained-release system [110].
	Carbamazepine	SSE	Orodispersible and immediate-release printlets [111].
**Parkinson’s** **disease**	Pramipexole	FDM	Stability and extended release [112].
	Levodopa	3D-printed polylactic acid (PLA) and chitosan (CS) neural tissue scaffolds	Extended drug release up to 14 days [46].
**Alzheimer’s** **disease**	Donepezil	Hybrid coated microneedles utilizing digital light processing (DLP) and SSE.	Enhanced skin permeation, sustained drug release, and biocompatibility [113].
	Curcumin	PLGA nanoparticles embedded in 3D-printed sodium alginate/gelatin scaffolds	Immediate sublingual delivery, controlled drug release over 18 days [114].

**Table 2:** 3D-printed multi-drug combinations for treating neurological disorders.

Neurological Condition	Drug Combination	3D Printing Technique	Key Benefits
**Epilepsy**	Levetiracetam + pyridoxine hydrochloride	Binder jet 3D printing	High-precision, multicompartmental dispersible tablets [116]
**Parkinson’s** **disease**	Levodopa+ benserazide+ pramipexole	Fused deposition modeling (FDM)	Floating mini-polypills with variable dosages, rapid and prolonged drug release [117].
	Levodopa+ carbidopa	Direct powder extrusion	Rapid drug release, suitability for hospital settings [118,119]

## Data Availability

No new data were created or analyzed during this study. Data sharing is not applicable to this article.
